# Supplementary material for: Global research priorities for COVID-19 in maternal, reproductive and child health: Results of an international survey
Source: PLoS One. 2021 Sep 24;16(9):e0257516. doi: 10.1371/journal.pone.0257516 (PMC8462675; doi:10.1371/journal.pone.0257516)
Supplement: S1 File — (DOCX) [file pone.0257516.s001.docx]

**S1 file - Questionnaire 1**

The Maternal, Newborn and Child Health (MNCH) Working Group of the COVID-19 Clinical Research Coalition would like to seek your opinion about priority research areas relating to COVID-19 and MNCH. We would be grateful for your answers to the questions in this short survey.

Thank you.

1. Your specialty:

- Obstetrics and Gynecology
- Pediatrics
- Sexual and Reproductive Health
- Midwifery and Nursing
- Infectious Diseases
- Public Health
- Epidemiology
- Other

2. Institution (including country):

3. Are you currently involved in research related to COVID-19? Y/N

a. If yes, what is your main area of focus?

4. Please select the five most urgent COVID-19 MNCH research areas from the list below

- Access to maternal, sexual and reproductive healthcare among vulnerable groups during the COVID-19 pandemic
- Access to healthcare for children during the COVID-19 pandemic
- Direct impact of COVID-19 on pregnant and infant populations
- Immunological mechanisms of resilience against COVID-19 among children
- Inclusion of pregnant women in COVID-19 vaccine trials

Inclusion of pregnant and breastfeeding women in COVID-19 treatment trials

- Indirect effects of the COVID-19 pandemic on pregnant and infant populations
- Infection prevention and control

Mental health sequelae of COVID-19 pandemic in pregnancy and postnatal periods

- Neurodevelopment of infants and young children exposed to COVID-19
- Pathophysiology and long term sequelae of pediatric inflammation and multisystem syndrome (PIMS-TS)
- Pathophysiological mechanisms of severe disease in children

Potential aerosolization of SARS-CoV-2 during second stage of labor

- Prevention and treatment strategies for pregnant women and newborns in humanitarian settings
- Safe breastfeeding
- Screening for SARS-CoV-2 during the antenatal, peripartum and postpartum periods
- Vertical transmission of COVID-19

6. Please provide any other MNCH priority research areas which were not included in the list

7. Any additional comments?
