# Supplementary material for: Global research priorities for COVID-19 in maternal, reproductive and child health: Results of an international survey
Source: PLoS One. 2021 Sep 24;16(9):e0257516. doi: 10.1371/journal.pone.0257516 (PMC8462675; doi:10.1371/journal.pone.0257516)
Supplement: S2 File — (DOCX) [file pone.0257516.s002.docx]

**S2 file - Questionnaire 2**

Thank you for agreeing to be contacted about the results of the MNCH Research Priorities questionnaire sent by the COVID-19 Clinical Research Coalition earlier this year.

We have reviewed the responses of the first questionnaire and identified the highest ranked research priorities. We would be grateful for additional feedback to refine these priorities further. Thank you.

1) Please rank the 10 research priorities from the list below (MOST important = 1, LEAST important=10)

1. Access to maternal, sexual and reproductive healthcare among vulnerable groups
2. Direct impact of COVID-19 on pregnant and infant populations
3. Indirect effects of the COVID-19 pandemic on pregnant and infant populations
4. Mental health sequelae of COVID-19 pandemic in pregnancy and postnatal periods
5. Access to healthcare for children during the COVID-19 pandemic
6. Infection prevention and control
7. Inclusion of pregnant and breastfeeding women in COVID-19 treatment trials
8. Vertical transmission of COVID-19
9. Prevention and treatment strategies for pregnant women and newborns in humanitarian settings
10. Screening for SARS-CoV-2 during the antenatal, peripartum and postpartum periods

2) Do you think these 10 priorities accurately reflect the MNCH research priorities in your region? Y/N

-If no, please provide details of additional research priorities which should be considered:

3) Are the research priorities understandable in their current format? Y/N

- If no, please provide comment:

4) Any additional comments? Y/N
