# Supplementary material for: Global research priorities for COVID-19 in maternal, reproductive and child health: Results of an international survey
Source: PLoS One. 2021 Sep 24;16(9):e0257516. doi: 10.1371/journal.pone.0257516 (PMC8462675; doi:10.1371/journal.pone.0257516)
Supplement: S3 File — (DOCX) [file pone.0257516.s003.docx]

**S3 file – Location (countries) of Questionnaire 1 respondents**

Afghanistan

Argentina

Belgium

Benin

Burkina Faso

Colombia

Costa Rica

Cuba

Democratic Republic of Congo

Honduras

India

Iraq

Japan

Madagascar

Malawi

Mali

Mexico

Niger

Pakistan

Peru

Saudi Arabia

South Africa

Sweden

Switzerland

Tunisia

United Kingdom

Unites States of America

Uganda

Venezuela
